# A systematic literature review of predicting patient discharges using statistical methods and machine learning

**DOI:** 10.1007/s10729-024-09682-7

**Published:** 2024-07-22

**Authors:** Mahsa Pahlevani, Majid Taghavi, Peter Vanberkel

**Affiliations:** 1 Department of Industrial Engineering, Dalhousie University, 5269 Morris Street, Halifax, B3H 4R2 NS Canada; 2 Sobey School of Business, Saint Mary’s University, 923 Robie, Halifax, B3H 3C3 NS Canada

**Keywords:** Discharge planning, Discharge prediction, Machine learning, Literature review, Regression, LOS

## Abstract

Discharge planning is integral to patient flow as delays can lead to hospital-wide congestion. Because a structured discharge plan can reduce hospital length of stay while enhancing patient satisfaction, this topic has caught the interest of many healthcare professionals and researchers. Predicting discharge outcomes, such as destination and time, is crucial in discharge planning by helping healthcare providers anticipate patient needs and resource requirements. This article examines the literature on the prediction of various discharge outcomes. Our review discovered papers that explore the use of prediction models to forecast the time, volume, and destination of discharged patients. Of the 101 reviewed papers, 49.5% looked at the prediction with machine learning tools, and 50.5% focused on prediction with statistical methods. The fact that knowing discharge outcomes in advance affects operational, tactical, medical, and administrative aspects is a frequent theme in the papers studied. Furthermore, conducting system-wide optimization, predicting the time and destination of patients after discharge, and addressing the primary causes of discharge delay in the process are among the recommendations for further research in this field.

Introduction

Healthcare demand and expenditures are increasing, putting pressure on healthcare systems [1]. Hospital operational and financial expenditures are affected by inpatient flow management. Effective patient flow involves preparing patients for each stage of care they require [2]. One of the most critical parts of inpatient flow is the process of patient discharge, often called Discharge Planning (DP). DP connects a patient’s hospital treatment and post-discharge care [3] and ensures continuity of care for patients when they leave the hospital.

Several studies have shown that a structured discharge plan may reduce hospital Length of Stay (LOS) and readmission rates while increasing patient satisfaction. Based on several studies, standard DP can improve patient outcomes including mortality/ survival rate [4–6], readmissions [5, 7–12], LOS [8, 12–14], and health-related quality of life [5, 8]. While there is little evidence that DP can lower healthcare expenditures [3, 15, 16], several studies indicate that proper DP results in cost savings for hospitals and the whole health system [17–20].

DP is a complicated procedure in hospitals that significantly impacts the entire healthcare system. As a result, numerous researchers have attempted to analyze this process, the causes of discharge delays, and its implications on the healthcare system. Several studies help this process by applying different methods to improve outcomes for both the system and the patients. They use approaches from the process improvement field, such as standardization of DP processes [16, 21–23], re-engineering of processes [24], applying knowledge management [25, 26], lean approaches [20, 27–31], and data analysis and quality assessment [32, 33] to examine the effectiveness of DP for patients moving from the hospital.

Combining patient-level data from Electronic Health Records (EHR) with advanced predictive tools can provide visibility into patient flow and discharge to help hospitals run more efficiently. Machine learning (ML) algorithms are effective in processing large amounts of data and provide a way to forecast the patients’ discharge elements in a timely, systematic, and accurate manner. With the growth in data analysis methods, several studies utilized different ML models to predict the discharge volume [34, 35], time [36, 37], and destination [38, 39]. These and other recent studies have looked into predicting the time and destination of discharged patients. However, we could not find a literature review article on this subject. This review article examines the prediction of discharge destination, which refers to where a patient is discharged (e.g., home, long-term care facilities), discharge time, which indicates when a patient is discharged (or LOS, which stands for the duration of a patient’s stay in a hospital). Additionally, it explores volume, which refers to the number of patient discharges in a fixed time period. These findings are reported in various sources, including journal articles, conference proceedings, grey literature, and books. Our discussion encompasses the evolution and contributions of developed methodologies in this field and summarizes the literature on discharge prediction.

The search strategy and identified articles are described in Section 2. Section 3 summarizes the use of predictive models in DP, including statistical-based and ML-based predictions. Section 4 summarizes the findings and recommends ways to improve DP and DP prediction in the future.

## Search strategy

Papers that met at least one of the following criteria are included in this review. (1) They investigated the prediction of DP factors from a statistical analysis. (2) They investigated prediction in DP problems utilizing ML models. The databases used are Scopus, Web of Science, Google Scholar, and Medline/Pubmed search engine. The authors discovered a group of relevant journal articles through scoping searches. These articles were then reviewed by an information specialist to generate a list of search phrases that encompassed each aspect of the review criteria, which was used to prepare the list of search terms. Search keywords in the title are Patient discharge planning; Discharge plan; Patient discharge prediction; Discharge time prediction; Patient discharge destination; Patient post-discharge; Post-discharge + long-term care; Post-discharge + home. All searches were carried out in August 2023, with a restriction on English-language publications and a period of 2002 to 2022.

### Search results

**Fig. 1:**
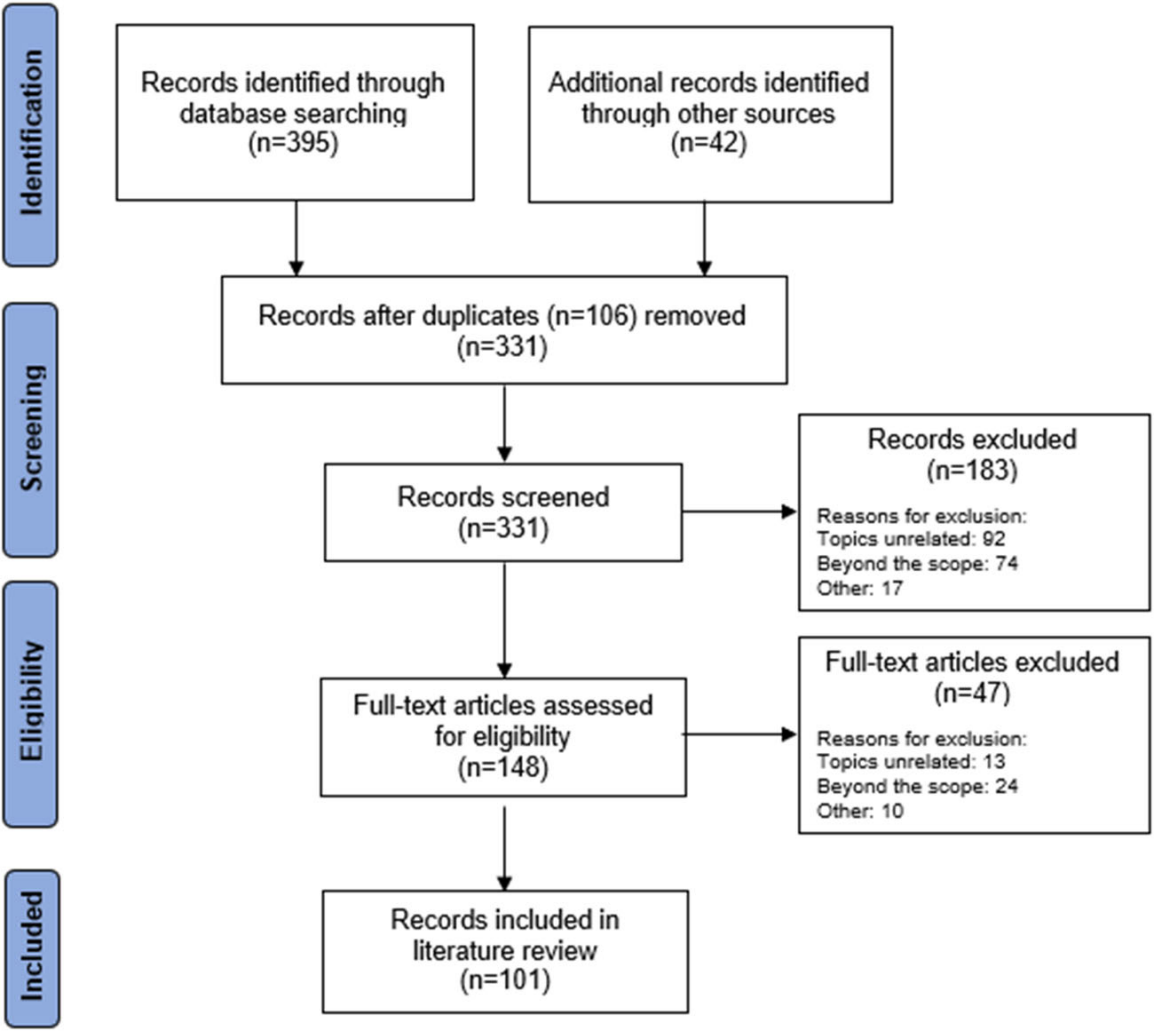
Literature search and screening flowchart

The Preferred Reporting Items for Systematic Reviews and Meta-Analyses (PRISMA) guidelines [40, 41] were followed in reviewing the papers. The search yielded a total of 437 studies, of which 106 were identified as duplicates, ultimately leaving 331 distinct papers. Articles that did not match the criteria based on their title and abstract were disregarded. The complete texts of the remaining articles were reviewed. 101 publications met one or both evaluation criteria (see Fig. 1).

### Classification of results

**Fig. 2:**
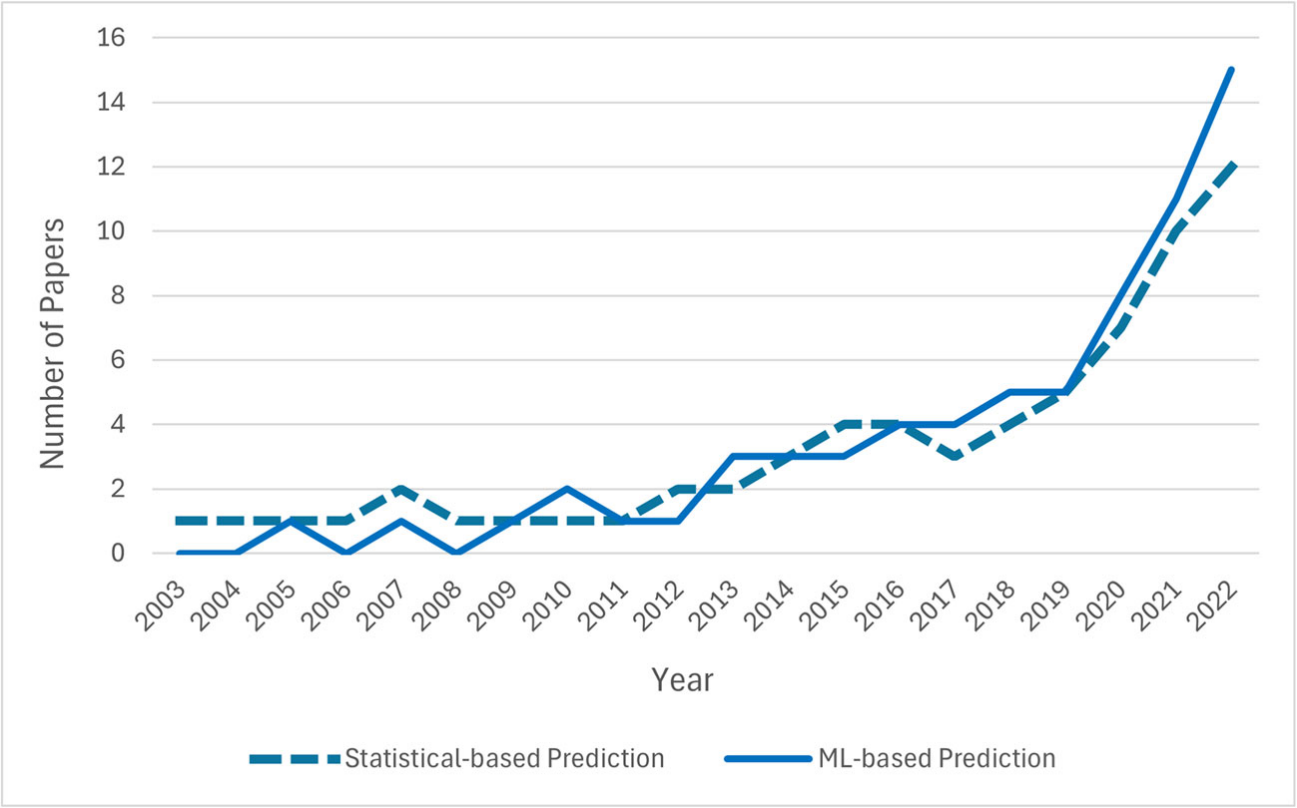
The trend of studies in discharge prediction during recent years

To demonstrate the breadth of this research area, we classified the articles into two categories based on their methodology. The objective of papers in both categories is the same: to predict the time or destination of patients after discharge. The first category of papers uses statistical methods (51 out of 101 papers), which is reviewed in Section 3.1. The second category of papers uses ML (50 out of 101 papers) and is reviewed in Section 3.2. The trends in these research areas are shown in Fig. 2, indicating the growing interest in understanding and improving the discharge process through prediction.

## Predicting discharge

Prediction is a strong tool for decision-making, from inventory management to strategic management [42]. Discharge prediction aims to improve inpatient flow by giving decision-makers accurate information [43, 44]. Furthermore, forecasting different aspects of DP, such as LOS, discharge time, and discharge destination, helps healthcare providers make better decisions for the entire system [45, 46]. This section reviews articles that determine and predict discharge factors using statistical tools (Section 3.1) and studies that use ML-based methods (Section 3.2).

### Statistical-based prediction

This subsection focuses on studies that analyze patient data with statistical methods to identify predictive factors related to discharge destination and discharge time. Most studies appear in clinical journals and seek the correlation between the discharge destination or time and patient factors such as demographic, socioeconomic, medical, etc. These studies use data analysis to determine the main features that can predict discharge destination and time.

#### Discharge destination prediction

Discharge destination is the most studied factor in this category. Knowing which characteristics impact the destination of patients is critical for physicians and hospital authorities [47, 48]. Based on their health situation, patients need to be discharged to either community-based places (e.g., home, home with support) or facility-based places (e.g., rehabilitation, long-term care).

One of the most studied groups of patients is orthopedic surgical patients. Studies help identify which patients may need additional care after surgical recovery. In several studies, different variables such as age, sex, race, socioeconomic factors, and family status are obtained as prediction factors for the next destination for patients [48–51].

Total joint arthroplasty (TJA) patients have attracted the attention of many researchers. TJA is a surgical procedure in which a damaged or diseased joint is replaced with an artificial joint or prosthesis. This procedure is commonly performed on the hip and knee. Since most of these patients need specialized care and assistance after surgery, defining their discharge destination is critical. Several studies find that demographics, clinical, and personal situations are the most important predictors of patients’ discharge destination after TJA [49, 52–58].

Mehta et al. [59] show that the level of community deprivation, representing the disadvantage or lack of resources within a community, can predict the discharge destination of patients undergoing hip arthroplasty. In a retrospective cohort study, Duque et al. [60] point to a connection between TJA performed under neuraxial anesthesia and an increased likelihood of home discharge. In a recently published study, to predict the discharge destination after total hip and knee arthroplasty, Hadad et al. [61] simultaneously investigate the performance of two tools; the preoperatively administered Predicting Location after Arthroplasty Nomogram (PLAN) and the postoperatively administered Activity Measure for Post-Acute Care (AM-PAC) “6-Clicks” basic mobility tools. They reveal that PLAN and “6-Clicks” basic mobility scores are well-performed predictors, suggesting that preoperative and postoperative variables influence discharge destination.

To investigate the impact of social support and psychological distress in the discharge plan after TJA, Zeppieri et al. [55] use the Risk Assessment and Predictive Tool (RAPT) (social support assessment) and modified STarT Back Tool (mSBT) (psychological distress assessment). Their results show that the RAPT is a proper tool to predict discharge destination. Focusing on the recent trends of community-based services, Cohen et al. [58] develop a modified RAPT score, which indicates the highest overall predictive accuracy of 92% and is capable of predicting home discharges.

Investigating spine surgery patients, Aldebeyan et al. [62] discover different demographic and clinical features that lead to facility-based discharge. They also use a multivariate logistic regression model to predict discharge destinations to other facilities rather than home. Through a retrospective cohort study focusing on the effect of age, Pennicooke et al. [63] show that patients over 70 had a higher chance of transferring to a facility-based destination. In another study, Lubelski et al. [64] create a calculator to estimate patients’ destinations after spine surgery. Their data analysis demonstrates that age, insurance type, marriage status, and surgical procedure are significantly associated with facility-based discharge destinations.

Kimmel et al. [48] develop a model to predict the facility-based destination for lower limb fracture patients. In another study, Glauser et al. [50] prove that the preoperative RAPT score is a highly predictive tool in lumbar fusion patients for discharge destinations that can predict admission to another facility or home. Using a multinomial logistic regression model, Ryder et al. [65] analyze and compare various characteristics and health outcomes of hospitalized patients with hip fractures. They also examine predictors of discharge destination to home or other facilities.

As patients have special needs after brain injury, many patients need to be discharged to a facility-based destination. To predict rehabilitation needs upon discharge after traumatic brain injury, De Guise et al. [66] consider different variables such as age, education, duration of posttraumatic amnesia, and clinical results. They find that having shorter posttraumatic amnesia lowers the chances of experiencing a disability and consequently lowers the need to be discharged to facility-based destinations. Focusing on the impact of race on the rehabilitation of traumatic brain injury patients, Oyesanya et al. [67] show that younger patients belonging to Latino or other racial/ethnic groups had a greater chance of being discharged to their homes rather than other facilities. In another study by Oyesanya et al. [68], sex and age are considered critical predictors for the discharge destination of traumatic brain injury patients. Also, using Logistic Regression (LR) on patients’ data, it is reported that younger and female patients have a lower chance of discharge to facility-based destinations.

Early prediction of post-stroke discharge destinations is found to be a way to improve patient outcomes, reduce costs, and improve the quality of care [69–72]. Also, some studies show that predicting and planning for a patient’s post-stroke discharge destination can reduce readmission rates, improve patient satisfaction, and increase the chance of successful rehabilitation [73–75]. Post-stroke discharge destination is typically predicted with clinical assessment and predictive modeling. Predictive modeling can be used to identify and analyze patient-specific predictors of post-stroke discharge destination. These predictors can determine which patients are more likely to be discharged to a facility-based or a community-based destination. Clinical assessment includes a patient’s medical history, current medical condition, and other factors such as age, sex, and comorbidities [76, 77].

Numerous studies reveal that patients’ physical situation, the family’s readiness at home, personal financial status, and marital status are significant predictive factors of discharge destination for patients after stroke [69, 71, 78–80]. A study by Nguyen et al. [81] reveals that marital status is crucial in determining discharge destination. However, immigrant and area-based socioeconomic status do not significantly impact discharge destinations. Moreover, Ouellette et al. [73] and Roberts et al. [82] propose that various functional and clinical outcome data at admission can be used to develop an accurate tool to predict discharge destinations for stroke patients. Kim et al. [83] establish a realistic assessment tool that forecasts home discharge for mild stroke patients after subacute rehabilitation therapy in tertiary institutions. This assessment tool considers a range of demographic, clinical, and functional variables as potential predictors. Cho et al. [75] investigate the link between the discharge status of post-stroke and patient characteristics using a probabilistic LR model. Based on their results, low readmission rates reflect complete care and proper discharge. Chevalley et al. [84] examine the effects of stroke patients’ socio-environmental characteristics and show that the most effective predictors of home discharge are living with others, receiving support at home, being married, and living at home before the stroke.

In another study, Gosling et al. [85] investigate the occurrence and risk factors associated with adverse discharge disposition (ADD) following cardiac surgery and present a tool to predict preoperative risks. Results show that patients with ADD are more elderly, female, have had a more extended hospital stay before surgery, and have undergone emergency surgery. Sex, race, payment type, injured region, physiologically base, and need for an Intensive Care Unit (ICU) are defined as determinant predictors of discharge destination for trauma patients by Lim et al. [86] and Strosberg et al. [87]. Hirota et al. [88] present two novel prediction models to determine where elderly patients with aspiration pneumonia will be discharged. They used various predictors, including age, sex, BMI score, and other clinical characteristics, to demonstrate that these models can aid in early-stage discharge planning. Table 1 presents an overview of the studies reviewed in this subsection, including the study name, prediction goals, patient populations, the method used, the main factors defined by studies as predictive factors, and dataset sizes.

**Table 1:** Studies related to Statistical-based discharge destination prediction

Study	Predicted parameter	Patient population	Methodology	Main factors	Dataset size
Agarwal et al. [76]	Discharge destination	Stroke patients	Logistic regressions	Age, sex, and the presence of premorbid social support	(n=104)
Lutz [78]	Facility-based discharges	Stroke patients	Grounded dimensional analysis	Functional Independence Measure (FIM) score, age, sex	(n=90)
Pablo et al. [49]	Discharge destination	Elective total hip replacement patients	Multivariate regression	walking ability, age, obesity	(n=1,276)
De Guise et al. [66]	Discharge destination	Traumatic brain injury patients	Logistic regressions	Age, education, Glasgow Coma Scale score	(n=339)
Nguyen et al. [81]	Discharge destination	Stroke patients	Multivariate logistic regression	Immigrant status, marital status	(n=326)
Lim et al. [86]	Discharge destination	Traumatic elderly patients	Multivariable random effect mixed model	Sex, race, payment type	(n=47,234)
Brauer et al. [70]	Community-based discharges	Stroke patients	Logistic regression	Admission functional status, age	(n=566)
Van der Zwaluw et al. [77]	Discharge destination	Stroke patients	Logistic regression	Cognitive dysfunction, age, BI score	(n=287)
Kimmel et al. [48]	Facility-based discharges	Lower limb fracture patients	Multivariable logistic regression	Age, proximal fracture type, fund source for the admission	(n=1,429)
West et al. [138]	Community-based discharges	Stroke patients	Behavioural mapping, statistical tests, multivariable median regression	Age, stroke severity, premorbid function	(n=73)
Stineman et al. [79]	Community-based discharges	Stroke patients	Logistic regression	Previous living circumstances, comorbidities, hospital course	(n=6,515)
Sharareh et al. [52]	Discharge destination	Joint arthroplasty patients	Cross-sectional analysis of different factors	Living statuses	(n=50)
Ouellette et al. [73]	Community-based discharges	Stroke patients	Logistic regressions and chi-square analyses	Health factors at the time of admission	(n=407)
Schwarzkopf et al. [139]	Discharge destination	Total hip arthroplasty patients	Multinomial regression	Race, insurance, morbidity	(n=14,326)
Halawi et al. [53]	Facility-based discharges	Joint arthroplasty patients	Multivariable logistic regression	Caregiver support, and patient expectation of discharge destination, age	(n=372)
Hansen et al. [57]	Facility-based discharges	Joint arthroplasty patients	RAPT, Binary logistic regression	Age, sex, health condition	(n=3,213)
Roberts et al. [82]	Discharge destination	Stroke patients	Receiver operator characteristic curve analysis, Linear regression	Functional status	(n=481)
Gholson et al. [54]	Community-based discharges	Joint arthroplasty patients	Multivariate logistic regression	Age, preoperative functional status, elective surgery status	(n=108,396)
Aldebeyan et al. [62]	Facility-based discharges	Lumbar spine fusion surgery patients	Multivariate logistic regression	Age, sex, comorbidities	(n=15,092)
Zeppieri et al. [55]	Discharge destination	Joint arthroplasty patients	RAPT, factorial analysis of variance	Social support, psychological distress	(n=231)
Dibra et al. [51]	Discharge destination	Revision joint arthroplasty patients	RAPT, Univariable logistic regression	Patient-reported discharge expectation	(n=716)
Sattler et al. [56]	Discharge destination	Knee arthroplasty patients	Univariable and multivariable logistic regression	Psychological, functional, and socio-demographic factors	(n=100)
Lubelski et al. [64]	Facility-based discharges	Spine surgery patients	Univariable and multivariable	Demographic variables, insurance status, baseline comorbidities	(n=257)
Ayyala et al. [39]	Facility-based discharges	Abdominal wall reconstruction patients	Multivariate logistic regression	Sex, history of diabetes, history of hypertension	(n= 4,549)
Glauser et al. [50]	Facility-based discharges	Posterior lumbar fusion patients	RAPT, Logistic regression	RAPT score, LOS, age	(n=432)
Kim et al. [30]	Community-based discharges	Moderate stroke patients	Logistic regression, weighted scoring model	Demographic, clinical, and functional factors	(n=732)
Mehta et al. [59]	Discharge destination	Hip arthroplasty patients	Adjusted binary logistic regression	Community area deprivation index level	(n=84,931)
Gosling et al. [85]	Facility-based discharges	Cardiac surgery patients	Stepwise backward logistic regression, used 5-fold and leave-one-out cross-validation	Age, sex, long LOS prior to surgery	(n=3,760)
Cohen et al. [58]	Discharge destination	Joint arthroplasty patients	RAPT, Multiple logistic regression	RAPT scores, demographic, and medical factors	(n=1,264)
Pennicooke et al. [63]	Discharge destination	Lumbar spine surgery patients	Multivariable nonlinear logistic regression	Age	(n=61,315)
Ryder et al. [65]	Facility-based discharges	Hip fracture patients	Multinominal logistic regression	Age, impaired cognition, reduced walking ability	(n=29,881)
Oyesanya et al. [67]	Discharge destination	Traumatic brain injury patients	Logistic regression	Race and ethnicity	(n=99,614)
Oyesanya et al. [68]	Discharge destination	Traumatic brain injury patients	Logistic regression	Age, sex	(n=221,961)
Hadad et al. [61]	Discharge destination	Joint arthroplasty patients	Regression models	Demographics, health factors	(n=11,672)
Hirota et al. [88]	Discharge destination	Aspiration pneumonia patients	Multilevel logistic regression	Age, sex, health factors	(n=34,105)

#### Discharge time prediction

Although most studies focus on the destination of patients after discharge, multiple investigations consider the time of discharge or equivalently a patient’s LOS at the hospital [89]. Using the RAPT and mSBT, Zeppieri et al. [55] show that lower social support leads to longer LOS after TJA. Also, Cohen et al. [58] develop a modified RAPT score which indicates the highest overall predictive accuracy of 92% and is capable of predicting LOS. Investigating spine surgery patients, Aldebeyan et al. [62] discover different demographic and clinical features that lead to an increase in the LOS. Through a retrospective cohort study focusing on the effect of age, Pennicooke et al. [63] show that patients over 70 had a higher chance of staying more in the hospital. In another study, Lubelski et al. [64] create a calculator to estimate patients’ LOS after spine surgery.

Hintz et al. [90] use LR models with time-dependent covariate inclusion to evaluate multiple models for predicting newborns’ time to discharge. They found that the prediction of discharge time is poor if only perinatal factors are considered, but it improves considerably with knowledge of later-occurring morbidities. Shukla and Upadhyay [91] investigate the factors influencing delay in discharge time for insured patients, considering discharge Turn Around Time. Predictors of same-day discharge following benign minimally invasive hysterectomy are identified by Alashqar et al. [92]. The demographic, surgical, and surgeon characteristics connected to discharge on surgical day 0 are examined using multivariate LR. They show that higher chances of same-day discharge are connected with robotic hysterectomy, quicker surgical duration, and minimum blood loss.

Moreover, in a recent investigation conducted by Lebruan et al. [93], the efficacy of the RAPT score in predicting LOS for patients undergoing TJA is examined. Unlike previous studies that considered total knee arthroplasty (TKA) and total hip arthroplasty (THA) together when analyzing the RAPT score, this research assesses them separately. The results reveal that THA patients outperformed TKA patients with similar RAPT scores, indicating a potential difference in RAPT performance between the two procedures. Table 2 demonstrates the studies reviewed in this subsection.

**Table 2:** Studies related to Statistical-based discharge time prediction

Study	Predicted parameter	Patient population	Methodology	Main factors	Dataset size
Hintz et al. [90]	Discharge time, LOS	Newborns patients	Linear and logistic regression	Clinical characteristics	(n=2,254)
Carter et al. [89]	LOS	Total knee replacement patients	Statistical tests	Age, sex, consultant	(n=2,130)
Aldebeyan et al. [62]	LOS	Lumbar spine fusion surgery patients	Multivariate logistic regression	Age, sex, comorbidities	(n=15092)
Shukla and Upadhyay [91]	LOS	General patients with insurance	Correlation and linear regression	Turn Around Time for insured patients	(n=443)
Zeppieri et al. [55]	LOS	Joint arthroplasty patients	RAPT, factorial analysis of variance	Social support, psychological distress	(n=231)
Lubelski et al. [64]	LOS	Spine surgery patients	Univariable and multivariable analyses	Demographic variables, insurance status, baseline comorbidities	(n=257)
Cohen et al. [58]	LOS	Joint arthroplasty patients	RAPT, Multiple logistic regression	RAPT scores, demographic, and medical factors	(n=1,264)
Alashqar et al. [92]	Discharge time	Benign minimally invasive hysterectomy patients	Multivariate logistic regression	Operative, and surgeon factors	(n=1,084)
LeBrun et al. [93]	LOS	Joint arthroplasty patients	RAPT scores, Multivariable analyses	BMI, Charlson comorbidity index, age	(n=18,000)

The studies discussed in these subsections employ statistical techniques to analyze historical data to predict the discharge destination and discharge time based on factors that are derived from the data. Many of these studies choose LR models for their analysis, depending on the specific variables, research question, and data characteristics. LR models the connection between a binary dependent variable and one or more independent variables. By examining historical data, these studies identify the primary predictors for discharge destination or discharge time, with demographic, socio-economic, and clinical factors being the main predictive elements.

RAPT is the other tool used in this area. The RAPT is a risk assessment tool that uses a set of risk factors, such as age, sex, medical status, and other patient characteristics, to calculate a risk score for each patient. The risk score is then used to categorize patients into defined classes. It should be noted that the accuracy of these prediction models is highly dependent on the quality of the data used to develop the models.

### ML-based predictions

ML can refer to circumstances in which machines can simulate human minds in learning and thus be used to solve problems [94]. Researchers in the healthcare sector have been applying artificial intelligence to aid better analysis and raise the efficacy of the entire healthcare industry [95]. Prediction modeling has experienced a tremendous rise in the popularity of techniques from the ML domains [96].

Multiple studies in recent years have looked into several models to predict discharge outcomes. The main outcomes investigated are the discharge destination, LOS and discharge time, and the discharge volume. These predictions can help hospitals and healthcare providers optimize bed utilization, manage staffing levels, and coordinate patient care more effectively. This subsection investigates studies in which researchers predict discharge outcomes using various ML models. The emphasis of these papers tends to be on comparing the performance of multiple ML models and, in some of them, on reporting the most important factors affecting discharge outcomes.

#### Discharge destination prediction

As mentioned earlier, we consider two possible destination types for patients after discharge from the hospital: community-based (e.g., home, home with support) and facility-based (e.g., rehabilitation, long-term care). Knowing whether patients are going to their homes or other facilities directly impacts discharge planning. Lack of capacity in other facilities can lead to extended hospital stays, increased risk of complications, and poorer health outcomes overall. In addition, it is a critical component for managing resources in a healthcare system [97, 98]. Researchers use a variety of ML models to predict discharge destinations based on historical data of patients; for example, Elbattah and Molloy [99] use different ML models to aid in planning senior care with a hip fracture focused on predicting discharge destination. They found that compared to other models, Random Forest (RF) offers significantly higher accuracy.

Considering various attributes of elective inpatient lumbar degenerative disc diseases after surgery, Karhade et al. [100] show that using different ML to develop an open-access web application to predict facility-based discharges has promising results. Lu et al. [101] introduce five ML models aimed at forecasting whether patients following knee arthroplasty can be discharged to their homes or require alternative facilities. The findings indicate that the extreme gradient boosting (XGB) model outperforms the remaining models. Furthermore, they identify key factors influencing the likelihood of facility-based discharges, including total hospital LOS, preoperative hematocrit, body mass index, sex, and functional status. Bertsimas et al. [102] use a wide range of ML models to predict various elements of patient flows, including discharge destinations using a unique patient representation. The findings show that EHR data combined with interpretable ML models can be leveraged to provide visibility into patient flows.

In another study for traumatic brain injury patients, Satyadev et al. [103] develop several ML models to predict discharge destination and propose the RF model as the best-performing model. Mohammed et al. [104] develop four different ML models (Gradient boosting (GB), RF, LR, Artificial neural networks (ANN)) to predict three discharge outcomes of patients after total knee arthroplasty, including discharge destination. The findings show that these ML models can predict the desired outcomes successfully.

Imura et al. [105] demonstrate that among three classification and regression tree models, the model including basic information, functional factor, and environmental attributes has the highest accuracy for classifying the likelihood of stroke patients being discharged at home. Imura et al. [106] also use ML to discover the relevant parameters influencing stroke patients’ home discharge who require a wheelchair after discharge. Consequently, the most closely connected variables for home discharge are revealed to be physical environmental characteristics of the patient’s home which may cause accessibility challenges. In a different investigation, Bacchi et al. [107] showcase the effective validation, both prospective and external, of ML models. These models utilize six variables to predict discharge-related information, particularly concerning home discharges for stroke patients.

Utilizing the XGB model, Ikezawa et al. [108] reveal that patients with ischemic cerebral infarction had excellent rates of home discharge when early nutrition occurred within the first three days of hospital admission. Morris et al. [109] develop a novel ML model called Bayesian additive regression trees that outperforms conventional regression analysis in predicting discharge destinations after trauma in elderly patients. Investigating the data set, they also find that age and the Glasgow Coma Scale upon admission play critical roles in predicting discharge destination. Mickle and Deb [110] also find that the XGB model can classify the discharge destination for patients in acute neurological care effectively, based on demographic and medical data available within 24 hours of their hospital admission.

In another study, to predict facility-based discharge destination after total knee arthroplasty, Chen et al. [111] apply ANN, RF, histogram-based gradient boosting (HGB), and k-nearest neighbor (KNN) on a large dataset. They discover that ANN and HGB have excellent predictive performance during internal and external validations and can perform well in distinguishing facility-based discharges. In a recent study by Geng et al., it is found that patients over 65, females, those with higher American Society of Anesthesiology scores, and those requiring more extensive fusion are more likely to be discharged to community-based care after elective anterior cervical discectomy and fusion.

The studies reviewed in this subsection are summarized in Table 3. The information in the table includes the predicted parameters and the target patient population. Additionally, the table lists the ML model(s) used for prediction, the best-performing ML models in studies where various models are employed, and the database size used.

**Table 3:** Studies of ML-based discharge destination prediction; the “*” denotes the best-performing model

Study	Predicted parameter	Patient population	Methodology	Main factors	Dataset size
Elbattah and Molloy [99]	Discharge destination	Elderly patients with hip fracture care	RF*, Boosted Decision Tree (BDT), NN, Linear regression		(n=2,000)
Karhade et al. [100]	Facility-based discharges	Elective lumbar degenerative disc disorders patients	NN*, BDT, SVM, Bayes Point Machine	Age, sex, BMI, fusion level, functional status	(n= 26,364)
Bacchi et al. [107]	Community-based discharges	Stroke patients	LR*, RF, DT, ANN	Age, sex, estimated pre-stroke mRS	(n= 2,840)
Lu et al. [125]	Facility-based discharges	Unicompartmental knee arthroplasty patients	Generalized linear model, RF, NN, XGB*	Total LOS, preoperative hematocrit, BMI, preoperative sodium	(n=7,275)
Bertsimas et al. [102]	Discharge destination	General patients	LR, CART DT*, Optimal Trees with Parallel Splits, RF, GBDT	Demographics, provider orders, diagnosis codes, medications	(n= 63,432)
Imura et al. [105]	Community-based discharges	Stroke patient	DT, Linear discriminant analysis, KNN*, SVM*, RF	Age, sex, stroke type	(n=481)
Satyadev et al. [103]	Discharge disposition	Traumatic brain injury patients	KNN, XGB, RF*	Vitals, demographics, mechanism of injury, comorbidities	(n=5,292)
Mohammed et al. [104]	Community-based discharges	Total knee arthroplasty patients	LR, GB*, RF*, ANN	Age, sex, race, admission month, admission on a weekend, admission type	(n=572,811)
Bacchi et al. [107]	Community-based discharges	Ischaemic or haemorrhagic stroke patients	LR, ANN*	Age, sex, stroke severity, health history	(n=1,158)
Ikezawa et al. [108]	Community-based discharges	Ischemic cerebral infarction patients	XGB	early nutritional initiation	(n=41,477)
Zhao et al. [133]	Facility-based discharges	Elective radical cystectomy patients	GBDT	Age, race, LOS, BMI	(n=11,881)
Morris et al. [109]	Discharge destination	Elderly patients with trauma	Bayesian additive regression trees	Age, comorbidities, LOS, physiologic parameters	(n=47,037)
Mickle and Deb [110]	Discharge destination	Acute neurological patients	LR, SVM, KNN, XGB*, RF	Age, glucose, admission weight	(n=5,245)
Chen et al. [111]	Facility-based discharges	Total knee arthroplasty patients	ANN*, RF, HGB*, KNN	LOS, age, BMI, sex	(n=434,550)
Geng et al. [137]	Facility-based discharges	Elective anterior cervical discectomy and fusion patients	RF	Age, Medicare insurance, American Society of Anesthesiology score, fusion levels	(n=2,227)

#### Discharge time prediction

The LOS and discharge time significantly affect capacity, costs, and patient satisfaction. By accurately forecasting the discharge time (or, equivalently, a patient’s LOS), hospitals can proactively address patient needs and improve their overall quality of care [112, 113]. Numerous studies use ML models to forecast the discharge time. By employing tree-based supervised ML models, Barnes et al. [36] demonstrate that early discharges are less predictable than midnight discharges. Their model surpasses clinicians in predicting daily discharges with greater accuracy and can effectively rank patients in order of proximity to upcoming discharges.

A clinically interpretable feedforward Neural Network (NN) model by Safavi et al. [114] helps to foresee which patients leave the hospital within 24 hours and their obstacles. The NN model finds clinical barriers, variations in clinical practice, and non-clinical factors among the 65 hurdles to discharge. In another study, Lazar et al. [37] design an RF model to predict the clinical preparedness for discharge in the next 24 to 48 hours. They find that this model predicts surgical discharges on a 48-hour basis with greater sensitivity than clinicians. Nemati et al. [115] use six different ML and statistical analysis models to predict the discharge time of COVID-19 patients to aid health professionals in making better decisions. After comparing the results, they find that the GB survival model performs better than the others.

Some studies predict LOS rather than discharge time. In recent years, inpatient LOS prediction has been studied using various ML models. To predict LOS, Liu et al. [116] apply Decision Tree (DT), Naive Bayesian (NB) classifiers, and feature selection models to a dataset from a geriatric hospital. They discover that using NB models to deal with the sizable amount of missing data can significantly improve the classification accuracy of forecasting LOS, particularly for the long-stay group. ANN model is also utilized by Gholipour et al. [117] to predict the LOS in ICU. They find that ANN outperforms the Lagrangian regression model. Tsai et al. [118] create an ANN model to predict the LOS for inpatients in a cardiology unit. The findings show that preadmission models can predict LOS and pre-discharge models.

Muhlestein et al. [119] devise a novel strategy for constructing a model that predicts LOS after craniotomy for a brain tumor. With high internal and external validation performance, an ML ensemble model predicts LOS and generates medical insights that could enhance patient outcomes. Bacchi et al. [120] look at how well ML models could estimate the likely LOS for stroke patients using admission data. According to this study, ML models may aid in prognosticating characteristics crucial to post-stroke DP. He et al. [121] develop an ANN-based multi-task learning model for the prediction of patient LOS. This model produces better results than single-task regression and classification models. By evaluating different ML models, Zhong et al. [122] demonstrate the RF and ANN models are accurate enough to predict the LOS of ambulatory total hip arthroplasty patients. A recently published study by Zeleke et al. [123] aims to develop and compare various ML models for predicting LOS and Prolonged LOS in general patient settings for those admitted through the emergency department. The objective is to create a framework for prediction rather than favoring a specific model. Eight regression models are developed for LOS prediction, with XGB regressions displaying the lowest prediction error. The studies reviewed in this subsection are summarized in Table 4.

**Table 4:** Studies related to ML-based discharge time prediction; the “*” denotes the best-performing model

Study	Predicted parameter	Patient population	Methodology	Main factors	Dataset size
Gholipour et al. [117]	LOS	Trauma patients	ANN*, Lagrangian regression	Mechanism of trauma, the site involved, vital signs and physical examination, laboratory findings	(n=125)
Barnes et al. [36]	Discharge time	General patients	Tree-based supervised ML models, Regression RF*	Admission and discharge times, demographics, basic admission diagnoses	(n= 8,852)
Elbattah and Molloy [99]	LOS	Elderly patients with hip fracture care	RF*, BDT, NN, Linear regression		(n=2,000)
Tsai et al. [118]	LOS	Cardiology patients	ANN*, Linear regression	Sex, age, location, main diagnosis	(n=2,377)
Turgeman et al. [112]	LOS	General patients	Regression tree	Demographics, outpatient and inpatient history, medication history, lab values and vital signs	(n=4,840)
Thompson et al. [135]	Prolonged LOS	Newborns	NB, Multi-layer Perceptron, Simple Logistic, SVM, DT, RF*, RT	Administrative data, minimal clinical data at the time of admission/birth	(n= 2,610)
Muhlestein et al. [119]	LOS	Brain tumor surgery patients	ML ensemble model	Nonelective surgery, preoperative pneumonia, sodium abnormality, race	(n= 41,222)
Kabir and Farrokhvar [113]	LOS	Surgical patients	ANN*, LR, SVM	surgical category	(n= 880,000)
Safavi et al. [114]	Discharge time	Surgical inpatients	Feedforward NN	Demographic, environmental, administrative, clinical	(n= 15,201)
Lazar et al. [37]	Discharge time	Surgical patients	RF	Age, sex, admission source, laboratory measurements, vitals	(n=10,904)
Bacchi et al. [107]	LOS	Stroke patients	LR, RF, DT, ANN*	Age, sex, estimated pre-stroke mRS	(n= 2,840)
Nemati et al. [115]	Discharge time	General patients	GB*, Fast SVM, Fast Kernel SVM	Age, sex	(n=1,182)
Bertsimas et al. [102]	Short-term discharges	General patients	Linear regression, CART DT, Optimal Trees, RF, GBT*	Demographics, provider orders, diagnosis codes, medications	(n= 63,432)
He et al. [121]	LOS, Flow	General patients	ANN	Age, sex, LOS, admit source department, medical features	(n=3,959)
Zhong et al. [122]	LOS	Ambulatory total hip arthroplasty patients	Multivariable LR, ANN*, RF*	Anesthesia type, BMI, age, ethnicity, white blood cell count	(n=63,859)
Gabriel et al. [136]	Discharge time	Surgical patients	Regression, RF*, balanced RF*, balanced bagging, NN, SVM	Patient’s surgical characteristics, age, sex, weight	(n=13,447)
Zeleke et al. [123]	LOS	General patients	Linear Regression, Ridge and Elastic-net regression, SVM, RF, KNN, XGB*	Demographic factors, mode of arrival/source of admission, risk categories, current problems	(n=12,858)

#### Other discharge outcomes prediction

Utilizing ML can also be a valuable tool for hospital practitioners and staff in determining several critical discharge planning outcomes. These results aid in predicting patient needs and optimizing the DP process. Morton et al. [124] examine the performance of several supervised ML models (i.e., multiple linear regression, support vector machines (SVM), multi-task learning, and RF) for predicting long LOS vs. short LOS in hospitalized diabetes patients. The results of this study show that the SVM model is the most promising for predicting short-term LOS. The number of discharges per day in hospital or discharge volume is another outcome that can be predicted using data. Knowing daily discharge volume in advance can diminish capacity-related uncertainties, leading to more optimized decisions regarding patient admission scheduling [34]. To predict daily inpatient discharges from the nephrology department, Luo et al. [125] use three models based on time series analysis. They discover that the RF model performs best.

The performance of a novel time-series ML model for predicting hospital discharge volume is compared to more straightforward models by McCoy et al. [35]. Their results emphasize that while more highly developed models are presented, time-series-based prediction can enhance clinical planning in the short term with little effort and without using big data sets, or computational power. Moreover, VanWalraven et al. [126] validate the Tomorrow’s Expected Number of Discharges model’s accuracy in predicting the number of hospital discharges the following day. Considering gynecologic oncology surgery patients, Lambaudie et al. [127] develop a prediction model including Classification and Regression Trees to determine who can stay at the hospital for less than two days. Levin et al. [128] address the support of multidisciplinary discharge-focused rounds problem using real-time EHR data and developing an ML-based discharge prediction model. Their findings show that computerized patient discharge predictions within multidisciplinary rounds help shorten hospital stays.

To help prioritize complex individuals and reduce healthcare inefficiency, Ghazalbash et al. [129] use classification ML models to predict multimorbidity using three indices. Results show the feasibility and utility of predicting multimorbidity status utilizing ML models, allowing early detection of individuals at risk of 30-day death and readmission. Moreover, three ML models are used in a study by Gramaje et al. [130] to forecast whether a patient after surgery should remain in the hospital or not. They offer intriguing results; while ML models in the class Remain show promising results, all ML models perform poorly in class Discharge. This study recommends including non-clinical characteristics of patients such as education, availability of family, finalized DP, and final physical examinations to boost the model’s performance.

Ahn et al. [131] investigate the discharge prediction and individual features of inpatients with cardiovascular diseases using five ML models. The XGB model outperforms other models. By assessing the outcomes of prediction models and visualizing simulated bed management, they also discover risk factors in cardiovascular patients and help hospital authorities develop resource management. Also, Gao et al. [132] predict inpatient discharges by proposing a novel ensemble deep learning model based on random vector functional links (edRVFL). Numerous forecasting indicators and statistical testing show that the suggested model surpasses the benchmark by a statistically significant margin. To improve DP for patients undergoing radical cystectomy, Zhao et al. [133] develop a Gradient Boosted Decision Tree (GBDT) model that supports patients’ complex conditions and helps them receive higher care. Jaotomboa et al. [134] compare the performance of different ML models on a hospital dataset to identify patients with prolonged LOS. By evaluating AUC, they demonstrate that among LR, classification and regression trees, RF, GB, and NN, the GB classifier outperforms the other models. The studies reviewed in this subsection are summarized in Table 5.

**Table 5:** Studies related to other ML-based discharge outcomes prediction; the “*” denotes the best-performing model

Study	Predicted parameter	Patient population	Methodology	Main factors	Dataset size
Ebell et al. [140]	Survival to discharge	Cardiopulmonary patients	Classification, Regression trees	Demographics, clinical features at admission	(n = 38,092)
Morton et al. [124]	Prolonged LOS	Diabetic patients	MLR, SVM*, SVM+, MTL, RF	Age, Sex, Race, Expected Primary Payer, Admission Type	(n= 10,000)
Luo et al. [125]	Daily discharges	Nephrology patients	Time series (ARIMA, LSTM and RF*	Demographics, discharge date	(n=1,091)
McCoy Jr et al. [35]	Discharge volume	General patients	Time-series		(n=101,867)
Van Walraven et al. [126]	Daily discharges	General patients	Survival tree approach	Age, sex, patient location throughout the admission	(n= 192,859)
Levin et al. [128]	Discharge rounds	General patients	Unit- specific models	Demographics, administrative, medications	(n= 12,470)
Ghazalbash et al. [129]	Multimorbidity status of patients	Old patients with discharge delay	Classification and regression trees, RF*, Bagging trees, XGB*, LR	Age, sex, marginalization, rural/urban residency, chronic conditions, LOS, admission type	(n=163,983)
Ahn et al. [131]	Discharge probability	Cardiovascular patients	XGB*, LR, RF, SVM, Multilayer perceptron	Demographics, administrative, medications	(n=572,811)
Gramaje et al. [130]	Discharge or Remain	Surgical patients	DT, RF, Bayesian Network*	Age, clinical conditions	(n=90)
Gao et al. [132]	Inpatient discharge	General patients	edRVFL		(n=417)
Jaotombo et al. [134]	LOS	General patients	LR, CART, RF, GB*, NN	Discharge destination, age, emergency admission, with more comorbidities notably mental health problems and dementia	(n=73,182)

This section explains the studies that explore the implementation of various ML models on a dataset and seek to predict discharge outcomes such as destination, time, volume, etc. However, just a few studies consider multiple discharge outcomes, such as destination, time, and volume. Another considerable gap among these studies is about the input data. Since integrating data from multiple sources can be a complex task, many studies are developed and validated using data from a single institution or a specific population, which might limit the generalizability of the results. Further research is needed to validate predictive models across diverse healthcare settings, populations, and geographic locations.

Moreover, most existing prediction models are based on historical data and may not fully use real-time data. Integrating real-time data, such as vital signs, laboratory results, and patient monitoring data, could enhance the accuracy of predictions. Limited use of advanced analytics techniques is found to be another gap in review studies. Although there are many different types of predictive modelling techniques, there has not been a lot of use of advanced analytics to predict patient discharge factors. Future studies can explore the application of advanced analytics to enhance predictions’ reliability and accuracy.

## Discussion

This paper presents a literature review focusing on studies that have employed prediction methods to estimate the destination, time, and volume of discharged patients. Numerous researchers have applied prediction methods to estimate different discharge factors using statistical and ML-based methods. Papers following the first approach (statistical methods in Section 3.1) aim to identify medical, demographic, and socioeconomic factors predicting patient discharge within specific cohorts. However, the second approach (ML-based models in Section 3.2) seeks to predict various discharge factors by implementing ML-based models on extensive datasets.

These two approaches share similarities but also exhibit differences. One of the significant distinctions is how they address discharge-related aspects. In the first approach, studies primarily focus on determining the destination and time of discharge. However, in the second approach, leveraging the enhanced capabilities of ML models, researchers can predict a more comprehensive array of variables such as daily discharge volume, discharge likelihood, and other related parameters.

In the first approach, discerning patient factors is para-mount, as studies endeavour to uncover influential variables for predicting discharge outcomes through statistical analyses. Conversely, ML-based studies may pinpoint significant factors, but the primary objective is not necessarily to isolate patient variables. Instead, their ultimate aim is to develop the most effective prediction model. In ML-based studies, the emphasis lies on comparing different models and identifying the one with the highest predictive accuracy.

This contrast is particularly evident when considering the methods employed. Unlike studies in the first approach, which utilize statistical methods, especially logistic regression, in the second approach, ML-based studies explore various ML models to minimize prediction errors. The statistical studies also try to customize their analyses for particular groups of patients, thereby enhancing the relevance of patient-related factors to predicted outcomes.

**Fig. 3:**
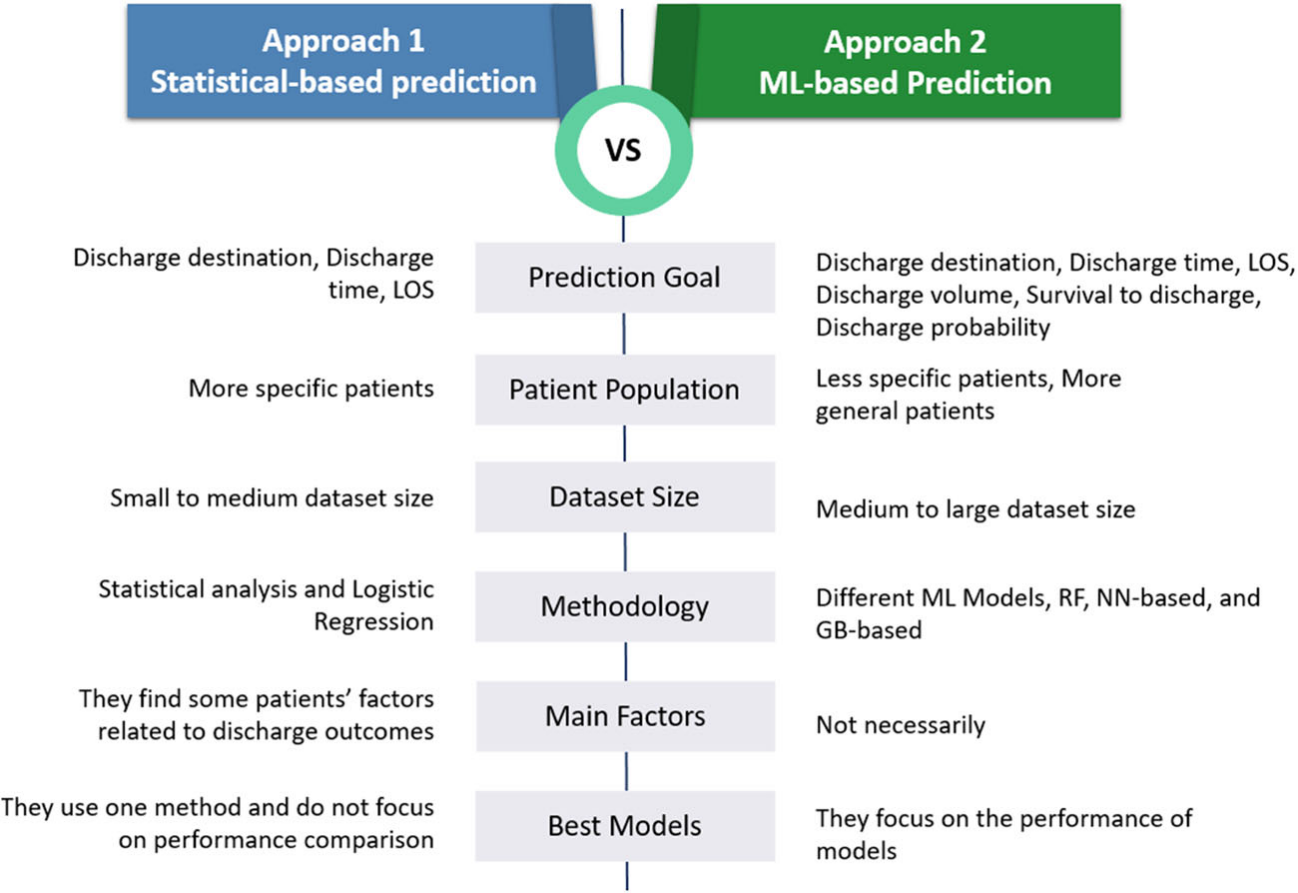
The characteristics of the two reviewed approaches

Figure 3 illustrates the characteristics of the two reviewed approaches. There are both similarities and differences between these approaches. However, the most noteworthy distinction lies in their methodologies, with additional notable variations. In the first approach, all the studies primarily investigate the prediction of destination and time of discharge, focusing on a specific group of patients. They aim to identify the most pertinent and critical patient factors related to discharge outcomes. Conversely, in the second approach, besides destination and time, other goals are considered. In this ML-based approach, studies are oriented toward comparing the performance of various ML models to determine the optimal model.

Most reviewed studies in Section 3.1 focus on orthopedic surgical patients, brain injury patients, and stroke. For orthopedic surgical patients, factors such as age, sex, race, socioeconomic factors, and family status are found to be significant predictors of discharge destination. Similarly, age, education, and clinical results are crucial for brain injury patients in predicting rehabilitation needs upon discharge. In stroke and cardiac surgery patients, factors such as patients’ physical condition, family readiness, financial status, and marital status play significant roles in determining discharge destinations. Also, several studies explore specific variables associated with discharge outcomes in other patient groups.

On the other hand, papers in Section 3.2 provide various ML models that utilize historical data. The chosen model is determined by the data set’s size, characteristics, and prediction type (whether a classification or a clustering model). A common application of ML-based models is in discharge destination prediction. By analyzing patient data, multiple studies utilize ML models to predict whether patients will be discharged to community-based or facility-based destinations. Another area where ML-based predictions have shown promise is in discharge time prediction. Accurately forecasting the discharge time allows hospitals to proactively address patient needs and improve patient flow and throughput. Moreover, ML-based predictions have been utilized to indicate discharge volume, enabling healthcare organizations to anticipate patient discharge outcomes. In most studies, time series models are utilized to predict discharge volumes. These approaches are effective in short-term forecasting and clinical planning without requiring extensive computational resources.

In terms of methodology, various methods are used to investigate statistical studies, such as LR and RAPT, with LR models being the most widely used. LR finds extensive application in both statistical-based and ML-based studies, although there are differences in their use and purpose. In statistical analysis, LR is primarily employed for inference, helping to understand the relationship between independent variables and binary outcomes. The emphasis here lies in comprehending the significance of each predictor. In contrast, in ML-based studies, LR is often utilized as a classification algorithm, predicting binary outcomes. The focus in this context shifts to predictive accuracy rather than inferential insights.

RF, NN-based, and GB-based models are the most commonly employed ML models in ML-based prediction studies. Among the 50 investigated studies, RF was used in 27, NN-based models in 20, and GB-based models in 17 for predictions. RF was the best-performing model in 13 studies [36, 37, 99, 103, 104, 122, 125, 126, 129, 135–137], making it the top-performing model in approximately 50% of its applications. NN models outperformed others in 11 studies [100, 106, 107, 111, 113, 114, 117, 118, 120–122], accounting for approximately 56% of their usage. GB models exhibited the best performance in 12 studies [104, 107, 108, 110, 111, 115, 123, 129, 131, 133, 134], establishing GB-based models as the best choice in 67% of their applications.

These models often outperform traditional statistical methods such as LR. It is important to note that the choice of models depends on the specific prediction task and the available data. Different ML models may suit diverse patient populations and discharge factors. Future studies can aim to validate and compare other models using more extensive and varied datasets, incorporate additional features such as non-clinical characteristics, and focus on improving the interpretability of ML models.

The researchers use various approaches to compare the performance of different ML models. One widely used performance metric is the area under the ROC curve. The ROC curve plots the true positive rate against the false positive rate for different classification thresholds. The AUC measures the overall performance of the ML model in distinguishing between positive and negative samples. Many studies use the AUC because it is easy to compute and interpret. Also, it provides a single value that summarizes the model’s overall performance, making it easier to compare the performance of different models on the same task or dataset.

Furthermore, several pieces of research focus on the destination, while others concentrate on the timing, daily discharge, or discharge volume. However, few studies examine multiple discharge patient outcomes, such as destination, LOS, volume, and clinical features. The lack of a diverse and generalized dataset is found to be another gap in this area. Incorporating input data from various healthcare institutions, populations, and locations, as well as considering real-time data, can increase the accuracy and validation of results. Another significant gap in this field is the application of prediction results as decision-making aids in hospital administration. Further effort is required to confirm the link between predictions, hospital actions, and quality of care. The incorporation of DP with other health facilities needs to be addressed for planning to be effective and precise in real-world scenarios.

Discharge is the final point of patient flow in the hospital, and for patients not discharged to home, it is linked to other healthcare facilities such as nursing homes, long-term care facilities, rehabilitation centers, etc. Accordingly, solutions to discharge concerns often lie outside the hospital and necessitate system-wide policies. Even in prediction studies, the majority of studies use a prediction tool to anticipate time or destination and assess the model’s effectiveness, and there is little discussion on the next steps. Another area of future research in this field is the practical use of the predictions to improve hospital processes and patient outcomes.

These problems need to be accurately modeled during the entire discharge process and predict system performance in a more realistic and detailed setting. While the DP problem presents itself as a difficult challenge, it also allows public health, healthcare systems, and hospitals to collaborate to develop best practices and intervention strategies. As a result, applying different tools, including data analysis, ML, operations research, and quality improvement, will benefit health administrators and patients.

## Glossary

**Table:** 

Acronym	Description
ADD	Adverse Discharge Disposition
ANN	Artificial Neural Networks
AUC	Area Under the ROC Curve
BDT	Boosted Decision Tree
BMI	Body Mass Index
DP	Discharge Planning
DT	Decision Tree
EHR	Electronic Health Records
FIM	Functional Independence Measure
GBDT	Gradient Boosting Decision Trees
HGB	Histogram-based Gradient Boosting
ICU	Intensive Care Unit
KNN	K-nearest Neighbor
LOS	Length of Stay
LR	Logistic Regression
ML	Machine Learning
MLR	Multinomial Logistic Regression
mSBT	Modified STarT Back Tool
MTL	Multi-Task Learning
NB	Naive Bayesian
NN	Neural Networks
RAPT	Risk Assessment and Predictive Tool
RF	Random Forest
ROC	Receiver Operating Characteristic
RT	Random Trees
SVM	Support Vector Machine
TJA	Total Joint Arthroplasty
THA	Total Hip Arthroplasty
TKA	Total Knee Arthroplasty
XGB	Extreme Gradient Boosting
